# Exploring Neurologists’ Perspectives: Barriers and Facilitators in Implementing Cognitive Care Planning

**DOI:** 10.1002/alz70858_103335

**Published:** 2025-12-25

**Authors:** Shaoqing Ge, Xaviera Xiao, Kevin H Sun, Bin Huang, Katherine Carroll Britt

**Affiliations:** ^1^ The University of Texas at Austin, School of Nursing, Austin, TX, USA; ^2^ BrainCheck Inc, Austin, TX, USA; ^3^ BrainCheck, Inc, HOUSTON, TX, USA; ^4^ BrainCheck Inc., Austin, TX, USA; ^5^ The University of Iowa College of Nursing, Iowa City, IA, USA

## Abstract

**Background:**

The aging global population is experiencing a rise in cognitive disorders like dementia, impacting individuals' independence and quality of life, and burdening families and healthcare systems. Addressing cognitive health in aging populations is a global health priority, yet few studies have examined the formal process of cognitive care planning (CCP) among neurologists.

**Method:**

This qualitative study used semi‐structured interviews to examine neurologists’ experiences, attitudes, and perceptions of implementing Cognitive Care Planning (CCP) in their practice. Participants were recruited from one medical center in Seattle, WA, using a purposive sampling approach. The interview guide was informed by the updated Consolidated Framework for Implementation Research (CFIR). From June to December 2023, we conducted audio‐recorded Zoom video conferencing interviews with seven neurologists. Deductive content analysis was employed to code interview data, deriving codes and subcodes from the pre‐existing CFIR framework.

**Result:**

The study identified several barriers to CCP integration, including limited treatment options, fear and shame associated with cognitive decline, complex health problems of patients, and patient willingness. Additional challenges included lacking technology integration, appointment structure, specialty‐specific demands on providers, organizational constraints like staffing and appointment time limitations, and a shortage of referral resources. Despite these barriers, neurologists also noted facilitators such as sufficient caregiver support, integrating a health champion, and making CCP appointments flexible and adaptable to patient needs.

**Conclusion:**

The study highlights challenges and opportunities in CCP implementation, aiming to inform strategies to improve cognitive health outcomes and quality of life for patients with cognitive impairments and their families.

